# Effect of Standardized Grape Powder Consumption on the Gut Microbiome of Healthy Subjects: A Pilot Study

**DOI:** 10.3390/nu13113965

**Published:** 2021-11-06

**Authors:** Jieping Yang, Patrick Kurnia, Susanne M. Henning, Rupo Lee, Jianjun Huang, Michael C. Garcia, Vijaya Surampudi, David Heber, Zhaoping Li

**Affiliations:** 1 Center for Human Nutrition, David Geffen School of Medicine, University of California, Los Angeles, CA 90095, USA; jiepingyang@mednet.ucla.edu (J.Y.); patrickt75@g.ucla.edu (P.K.); shenning@mednet.ucla.edu (S.M.H.); rupolee@mednet.ucla.edu (R.L.); jianjunhuang@mednet.ucla.edu (J.H.); mcgarcia@mednet.ucla.edu (M.C.G.); vsurampudi@mednet.ucla.edu (V.S.); dheber@mednet.ucla.edu (D.H.); 2 Department of Medicine, VA Greater Los Angeles Health Care System, Los Angeles, CA 90073, USA

**Keywords:** grape powder, gut microbiome, cholesterol, bile acids, healthy adults

## Abstract

Grapes provide a rich source of polyphenols and fibers. This study aimed to evaluate the effect of the daily consumption of 46 g of whole grape powder, providing the equivalent of two servings of California table grapes, on the gut microbiome and cholesterol/bile acid metabolism in healthy adults. This study included a 4-week standardization to a low-polyphenol diet, followed by 4 weeks of 46 g of grape powder consumption while continuing the low-polyphenol diet. Compared to the baseline, 4 weeks of grape powder consumption significantly increased the alpha diversity index of the gut microbiome. There was a trend of increasing Verrucomicrobia (*p* = 0.052) at the phylum level, and a significant increase in *Akkermansia* was noted. In addition, there was an increase in *Flavonifractor* and *Lachnospiraceae_UCG-010*, but a decrease in *Bifidobacterium* and *Dialister* at the genus level. Grape powder consumption significantly decreased the total cholesterol by 6.1% and HDL cholesterol by 7.6%. There was also a trend of decreasing LDL cholesterol by 5.9%, and decreasing total bile acid by 40.9%. Blood triglyceride levels and body composition were not changed by grape powder consumption. In conclusion, grape powder consumption significantly modified the gut microbiome and cholesterol/bile acid metabolism.

## 1. Introduction

Grapes not only contain various phytochemicals, such as catechins, proanthocyanidins, anthocyanins, leucoanthocyanidin, quercetin, kaempferol, stilbenes, ellagic acid, and hydroxycinnamates, but are also a good source of fiber [1,2,3]. The antioxidant, antibacterial, and antiviral effects of grapes, grape extract, or grape phenolic compounds from grapes have been previously reported [3]. Eight weeks of dietary raisin consumption was found to ameliorate liver function and atherosclerotic lesion formation in rabbits fed an atherogenic diet (0.5% cholesterol) [4]. A recent mouse study showed that table grape consumption can decrease adiposity and improve markers of hepatic steatosis, and is associated with an improvement in the gut microbiome [5].

An altered intestinal gut microbiota, i.e., dysbiosis, has been associated with the development of metabolic diseases, such as obesity, type 2 diabetes mellitus (T2DM), and cardiovascular disease [6]. Growing evidence has suggested the crucial role of the gut microbiome in host cholesterol homeostasis, including, but not limited to, microbial cholesterol and bile acid (BA) metabolism [7,8].

Studies have shown that fruits high in polyphenols can have prebiotic effects, leading to changes in gut microbiota composition. [9]. Also, our prior studies in mice demonstrated that the supplementation of an obesogenic diet with a polyphenol-rich pomegranate extract decreased blood cholesterol levels and liver cholesterol content. In addition, a combination of pomegranate extract and inulin fiber further decreased cholesterol synthesis, increased cholesterol degradation, and increased cholesterol and BA excretion, in association with changes in the gut microbiota [10].

A previous mouse study demonstrated that dietary grape seed extract supplementation improved blood lipid profiles by attenuating hepatic cholesterol synthesis, enhancing BA biosynthesis, decreasing lipogenesis and intestinal BA transportation, as well as increasing fecal BA and lipid excretion [4,5,11].

Grapes are one of the most commonly consumed fruits, but there is limited information regarding the effects of grape consumption on the gut microbiome and cholesterol metabolism in humans. The present pilot study was designed to assess if daily consumption of two servings of California grapes in the form of whole grape powder (one dose of 46 g) alters the intestinal microbiota, host cholesterol, and BA metabolism in healthy free-living individuals.

## 2. Method and Materials

### 2.1. Study Design

**Figure 1:**
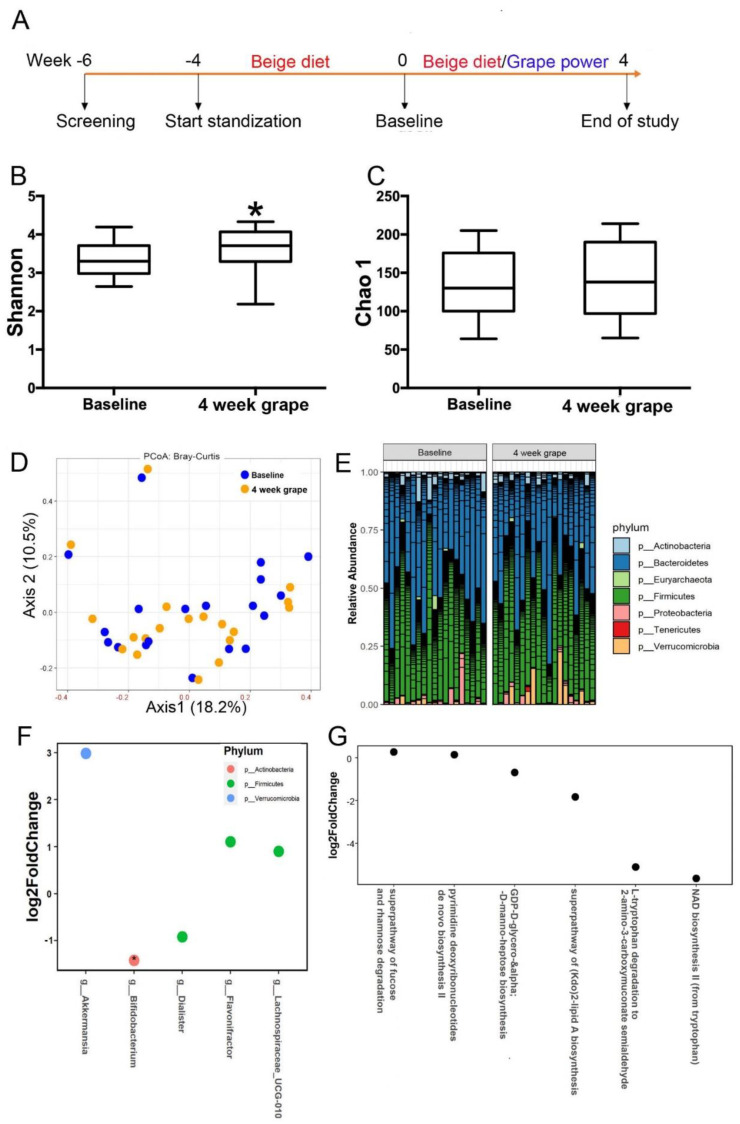
Impact of 4-week grape powder intake on gut microbiota. Microbiome diversity analysis. (**A**) Study design. Alpha diversity analysis of fecal microbiota, (**B**) Shannon index and (**C**) Chao index. * *p* < 0.05. (**D**) Principal coordinate analysis plot of beta diversity measure Bray–Curtis dissimilarity. (**E**) Fecal microbial profile at phylum level. (**F**) Fecal microbial genera identified to be significantly different in abundance between baseline (week 4) and 4-week grape powder intake (week 8) using DESeq2. * adjust *p* < 0.05. (**G**) Fecal microbial metabolic pathways were identified to be significantly different between the baseline and 4-week assessments using DESeq2, *p* < 0.05.

This study was carried out at the Center for Human Nutrition, University of California, Los Angeles, California, USA and was registered at clinicaltrials.gov as NCT05025189. The clinical protocol was approved by the Internal Review Board of the University of California, Los Angeles All subjects gave written informed consent before enrollment in the study. This is a two-phase intervention studyincluding a 4-week standardization phase and 4-week intervention phase. After 4 weeks of consuming a low-polyphenol and low-fiber diet (fiber <10 g and polyphenol-rich fruits/vegetables <3 servings per day) [12], healthy free-living subjects consumed one dose (two servings) of standardized freeze-dried whole table grape powder (46 g) daily for 4 weeks while continuing to consume the low-fiber and low-polyphenol diet (Figure 1A). Eligibility criteria included good health, 18 to 55 years of age, and habitually consuming a low-fiber/low-polyphenol diet (Table 1). Postmenopausal women and subjects taking any medication or dietary supplement that could interfere with the absorption of polyphenol were excluded. In addition, subjects taking antibiotics, laxatives or probiotics within the past 3 months or those who had sensitivity to grapes were excluded. The composition of the grape powder used in this study is provided in Tables S1 and S2. Fasting blood and urine samples were collected and body composition recorded at week 0 (the beginning of the standardization), week 4 (before grape powder supplementation) and week 8 (end of grape powder supplementation). Stool samples were collected at week 4 and week 8. At weeks 0, 4 and 8, the subjects completed and returned 3-day food records that were evaluated by a dietitian for compliance with the low-fiber/low-polyphenol diet [12].

### 2.2. Fecal 16S rRNA Gene Sequencing and Taxonomic Analysis 

DNA extraction and sequencing of the 16S rRNA gene were performed as previously described by UCLA Microbiome Core [13]. In brief, fecal bacterial DNA was extracted using the QIAGEN PowerSoil kit with bead beating. The V4 region of the 16S gene was amplified and barcoded using 515f/806r primers then 250 × 2 bp sequencing was performed on an Illumina MiSeq. Amplicon sequence variants (ASVs) were identified using DADA2 and annotated against the SILVA v138 database. Alpha diversity metrics (Chao1 and Shannon index) were calculated after rarefication to a depth of 50168 sequences per sample and tested for significance using the Wilcoxon signed-rank test at *p* < 0.05. Beta diversity was calculated using Bray–Curtis dissimilarity. The relationships of samples across groups were determined by permutational multivariate analysis of variance (PERMANOVA) using the Adonis command provided by Vegan in R and were displayed via principal coordinate analysis (PCoA) ordination [14]. DESeq2 was used to identify abundance changes at the genus level and differences that occurred between the baseline, week 4 samples, and week 8 samples following the grape powder intervention. A statistical model (formula: ~ subject + treatment) was passed to DESeq2, which permitted accounting for differences between subjects while estimating the effect due to the treatment [12,14]. *p*-values were adjusted for multiple testing using Benjamini–Hochberg false discovery rate correction in DESeq2. As Bonferroni correction is often considered overly conservative, we listed genera with *p*-value < 0.05 and marked those with adjusted *p* <0.05 using *. The PICRUSt2 was used to predict the function of the gut microbiota on metabolic pathways in the MetaCyc database from 16S rRNA gene amplicon sequencing [15]. DESeq2 was used to identify the predicted functional pathways significantly different between baseline and intervention.

### 2.3. Serum Lipid Measurement 

Serum total triglycerides (TG), total cholesterol (TC), high-density lipoprotein cholesterol (HDL-C), and low-density lipoprotein cholesterol (LDL-C) were measured spectrophotometrically using established cholesterol and triglyceride analysis reactions (Pointe Scientific, Canton, MI, USA) [10].

### 2.4. Serum Bile Acid (BA) Measurement

100 µL serum was precipitated with 500 µL ice-cold methanol, vortexed and centrifuged at 10,000× *g* for 15 min. The supernatant was dried using SpeedVac evaporator and then resuspended in 100 µL 50% methanol/H_2_O (1:1 v/v) for analysis. Cholic acid (CA), chenodeoxycholic acid (CDCA), deoxycholic acid (DCA), lithocholic acid (LCA), ursodeoxycholic acid (UDCA), hyodeoxycholic acid (HDCA), taurocholic acid (TCA), taurochenodeoxycholic acid (TCDCA), taurodeoxycholic acid (TDCA), taurolithocholic acid (TLCA), glycocholic acid (GCA), glycochenodeoxycholic acid (GCDCA), glycodeoxycholic acid (GDCA), taurolithocholic acid (TLCA), glycodeoxycholic acid-d4 (GDCA-D4) and chenodeoxycholic acid-d4 (CDCA-D4) were purchased from Cayman Chemical (MI, USA). The concentration of GDCA-D4 and CDCA-D4 was used as internal standard. Liquid chromatography coupled to electrospray ionization and triple quadrupole mass spectrometry (LC-ESI-MS/MS) at negative mode was used for BA analysis. Zorbax SB-C18 (150 × 2.1 mm; 5 µm) was used for separation. Five percent acetonitrile in methanol was used as mobile phase A, and 7.5 mM ammonium acetate in H2O, adjusted to pH 4.0 using acetic acid was used as mobile phase B at a total flow rate of 0.2 mL/min [16].

### 2.5. Statistical Analyses 

The sample size was calculated using data from a published intervention of 10 healthy volunteers consuming red wine, which is derived from grapes [17]. The wine intervention was associated with an increase in bifidobacteria from 7.1 ± 2.3 log10 copies/g feces (control) to 9.9 ± 1.8 log10 copies/g feces (red wine) providing an effect size of 1.35. Based on that data, we have >90% power to detect an expected increase in bifidobacteria with grape consumption, with a sample size of 18 subjects. A paired t-test or Wilcoxon signed-rank test were used to compare the change in outcome for serum lipid and BA concentration after intake of grape powder compared to placebo. Descriptive analysis, mean (SD) and statistical analysis were performed using the Statistical Package for the Social Sciences™ (SPSS) version 8.0 software (SPSS Inc., Chicago, IL, USA).

## 3. Results

### 3.1. Study Subjects

This was a two-phase intervention study, including a 4-week standardization phase and a 4-week intervention phase. A total of 21 healthy subjects were screened; one dropped out during phase 1, and another did not finish the study. Data from 19 subjects, aged 21–55 years, were included in the statistical evaluation. No adverse effects were reported. BMI and body weight were similar between the baseline and the 4-week grape powder intake (164.8 (47.7) vs. 165.0 (49.4)) (Table 1).

### 3.2. Effect of Grape Powder Intake on Gut Microbiota

Four weeks of grape powder intake significantly increased the alpha diversity index (Shannon, *p* = 0.04), but not Chao1, when compared to the baseline (Figure 1B,C). The beta diversity measure Bray–Curtis dissimilarity was calculated and visualized via principal coordinate analyses (PCoA). No distinct separation between the baseline and the 4-week grape powder intake was observed (Figure 1D). Comparing the 4-week grape powder intake to the baseline, the fecal relative abundance of the phylum Verrucomicrobia (Table 2 and Figure 1E) and three genera, *Akkermansia, Flavonifractor,* and *Lachnospiraceae UCG-010* (Figure 1F), was significantly increased, while the relative abundance of two genera, *Bifidobacterium* and *Dialister* (Figure 1F), was significantly decreased after grape powder intake. A total of 357 metabolic pathways were predicted by PICRUSt2 (data not shown). A total of 6 out of 357 pathways were significantly different between the baseline and the 4-week grape powder intake (Figure 1G). The fucose and rhamnose degradation pathway, and the pyrimidine deoxyribonucleotides de novo biosynthesis II pathway, were significantly increased by the grape intervention. The (Kdo)2-lipid A biosynthesis, NAD biosynthesis II (from tryptophan), L-tryptophan degradation to 2-amino-3-carboxymuconate semialdehyde, and GDP-D-glycero-alpha-D-manno-heptose biosynthesis pathways were downregulated (Figure 1G).

### 3.3. Grape Powder Intake and Serum Lipids and Bile Acids 

**Figure 2:**
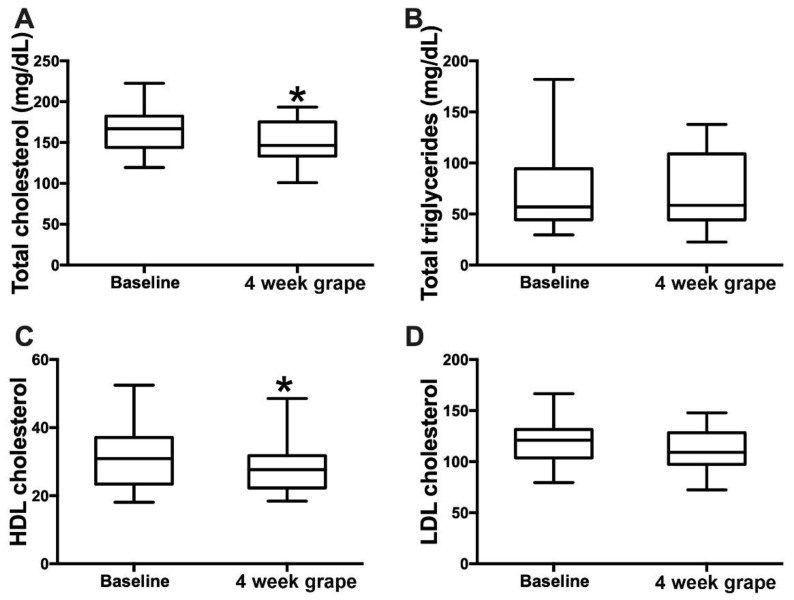
Effects of 4 weeks’ grape powder intake on serum (**A**) total cholesterol, (**B**) total triglycerides, (**C**) high-density lipoprotein (HDL) cholesterol and (**D**) low-density lipoprotein (LDL) cholesterol. * *p* < 0.05.

After 4 weeks of grape powder consumption, the serum total cholesterol had decreased by 6.1% (baseline: 164.8 mg/dL (27.3); intervention: 154.7 mg/dL (25.1); *p* = 0.04) and HDL cholesterol by 7.6% (baseline: 31.4 mg/dL (9.4); intervention: 29.0 mg/dL (8.0); *p* = 0.01). LDL cholesterol showed a decreasing trend by 5.9% (baseline: 118.6 mg/dL (23.5); intervention: 111.6 mg/dL (19.6); *p* = 0.08), and triglyceride levels were not changed (Figure 2). In addition, 4 weeks of grape powder consumption induced a significant decrease in the serum conjugated primary bile acids (BAs) GCGCA and TCDCA by 47.9% and 42.1%, respectively, and the conjugated secondary BAs GDCA and TDCA by 27.8% and 35.8%, respectively (Table 3).

### 3.4. Correlation between Gut Microbiota and Serum Lipids and BAs

**Figure 3:**
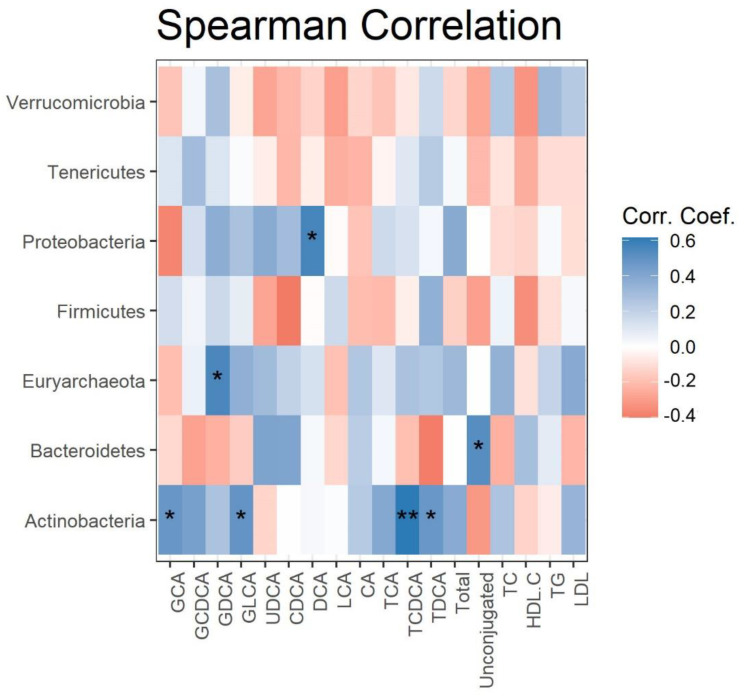
Correlation of fecal phyla with serum BAs. Heatmap depicting the Spearman correlation patterns (* *p* < 0.05, ** *p* < 0.01) of changes in relative abundance of fecal bacterial phyla and concentrations of serum BAs over 4 weeks’ grape powder intake.

Spearman correlations were calculated and plotted as a heatmap between the changes in serum lipids, BA levels, and gut microbes at the phylum level, after 4 weeks of grape powder intake (Figure 3). The changes in the relative abundance of *Proteobacteria*, *Euryarchaeota,* and *Bacteroidetes* were positively correlated with the changes in serum DCA, GDCA, and the ratio of unconjugated BAs to total Bas, respectively. The changes in the relative abundance of *Actinobacteria* were positively correlated with four out of seven conjugated BAs (GCA, GLCA, TCDCA, and TDCA). No significant correlation was detected between the changes in serum triglycerides, cholesterol, HDL cholesterol, LDL cholesterol, and microbial phyla (data not shown).

## 4. Discussion

This study aimed to test the hypothesis that grape powder consumption leads to changes in intestinal microbiota, host cholesterol, and BA homeostasis. The data presented demonstrate that the consumption of grape powder for 4 weeks was associated with changes in the gut microbiota and a decrease in blood cholesterols (total, HDL, and a trend of decreasing LDL cholesterol). There was also a decrease in conjugated BAs. In the present study, no significant differences in body composition, or adverse digestive symptoms were observed between the baseline (week 4) and the end of the grape intervention (week 8, data not shown), suggesting that the daily consumption of 46 g of whole grape extract powder was safe and well tolerated in healthy subjects. 

We observed a significant increase in the Shannon index (microbial richness and evenness) at the end of the 4-week grape intervention compared with the baseline, supporting the potential of daily grape powder consumption to change the gut microbiome in free-living healthy subjects consuming a low-fiber/low-polyphenol diet. In a prior study, alpha diversity, by Shannon index, has been positively correlated with dietary quality, including the intake of fruits and vegetables, high-fiber whole-grain products, and fish in overweight and obese pregnant women [18].

In summary, our data suggest that grape powder intake potentially improves the low alpha diversity phenotype of free-living healthy subjects consuming a low-fiber/low-polyphenol diet. We also observed that grape powder induced a significant increase in *Akkermansia* abundance. *Akkermansia* degrades mucin, which stimulates goblet cells to make more mucin, resulting in increased protection of the gut barrier, which, in turn, helps to prevent dysbiosis [19]. Emerging evidence suggests that *Akkermansia* is also involved in an interaction between gut epithelium and the microbiota in diet-induced obesity [20]. A recent study demonstrated that oral supplementation with *Akkermansia* improved glucose and lipid metabolism in overweight and obese subjects [21]. The identification of dietary strategies to increase *Akkermansia* abundance in the gastrointestinal tract is of great interest. Therefore, the observation that 4 weeks of grape powder consumption significantly increased *Akkermansia* abundance is significant. The abundance of *Bifidobacterium,* however, decreased significantly after grape powder intake. A previous study showed that grape polyphenol greatly stimulated *Bifidobacterium* growth ex vivo [22]. It is noteworthy that the grape powder extract used in this study included nutrients of whole grape, including phenolic compounds, fiber, and sugar. In this study, 46 g of the grape powder used provided about 163 mg of phenolic compounds and about 35 g of sugar (Supplementary Table S2) [23]. Whether other components, such as sugar, in grape powder reduced the abundance of *Bifidobacterium* is unknown and requires further investigation.

The other two genera with significantly increased abundance after grape powder intake were *Lachnospiraceae UCG-010* and *Flavonifractor.* There is limited information on the health impact of *Lachnospiraceae UCG-010* and *Flavonifractor*. *Flavonifractor plautii* (FP) has been shown to be involved in microbial catechin metabolism and to affect host immunity [24,25]. The increase in *Flavonifractor abundance* likely resulted from the catechin content of grape powder. The physiological role of *Lachnospiraceae* is largely unknown and remains controversial. *Lachnospiraceae UCG-010* has previously been shown to be positively correlated with intestinal inflammation in children with cystic fibrosis [26].

During the 4-week grape powder intervention, no adverse GI effects were reported using weekly digestive symptom logs (data not shown). Previous studies also support the anti-inflammatory capacity of grapes or grape phytochemicals in the GI tract. Further studies are needed to understand the interaction between diets and the microbiome in host physiology, in terms of health and disease.

Increased blood cholesterol levels are an established risk factor for cardiovascular diseases, while increased HDL cholesterol levels are associated with reduced risk and cholesterol excretion. The liver removes cholesterol from the circulation via the secretion of bile, which includes cholesterol and bile acids synthesized in the liver [27]. HDL functions to transport excess cholesterol to the liver, where it is converted into Bas [28,29]. The liver and gastrointestinal tract, via enterohepatic circulation, determine the size of the body cholesterol pool, which influences the blood cholesterol levels and whole-body cholesterol homeostasis.

Dietary intervention is the primary approach to cholesterol management [30]. An epidemiological study suggests that soluble fiber, 2–10 g daily, is associated with small, but significant, decreases in total cholesterol and LDL cholesterol [31]. In the present study, 46 g of grape powder provided about 2–3 g of dietary fiber (Supplementary Table S2) [23]. We observed a decrease in circulating total cholesterol by 6.1%. However, HDL cholesterol was also reduced by 7.6% after consuming grape powder for 4 weeks. Low-fat diets are known to decrease HDL cholesterol levels by affecting HDL metabolism [32,33]. A previous study showed that sugar intake was negatively associated with HDL cholesterol in US adults. Unfortunately, this study was designed to evaluate the grape powder intake in healthy free-living individuals, and sugar intake was not controlled in the study subjects. Therefore, whether the sugar content of grape powder resulted in the HDL cholesterol reduction requires further investigation. Further studies are needed to evaluate the health impact of a decrease in both cholesterol and HDL cholesterol in metabolically healthy, free-living individuals following dietary intervention.

In addition, grape powder consumption for 4 weeks significantly decreased blood BA levels, particularly conjugated BAs (taurine and glycine conjugation). Whether consuming grape powder changed hepatic BA conjugation, or conjugated BA transport between organs, remains unknown.

In summary, our results provide novel preliminary information about the effects of grape intake on the gut microbiome, host cholesterol, and BA metabolism. These data will assist in the future design of studies to explore the health benefits of grape consumption.

## Figures and Tables

**Table 1:** Demographics of study participants (*n* = 19).

	Baseline	4-Week Grape
Age	33.5 (10.5)	
Sex, % women	70%	
BMI (kg/m^2^)	28.5 (10.1)	28.5 (10.3)
Weight (lb)	164.8 (47.7)	165.0 (49.4)

Values are means (SDs) (*n* = 19).

**Table 2:** The relative abundance of phyla (percent) at baseline and end of 4-week grape powder consumption.

	Baseline	4-Week Grape
Bacteroidetes	45.34 (18.08)	40.69 (18.41)
Firmicutes	47.34 (16.42)	51.39 (16.82)
Actinobacteria	3.71 (3.68)	2.49 (2.02)
Verrucomicrobia	0.5 (1.02)	3.54 (6.27) *
Proteobacteria	2.54 (5.02)	1.5 (1.49)
Euryarchaeota	0.52 (1.4)	0.26 (0.65)
Tenericutes	0.05 (0.13)	0.13 (0.48)

Values are means (SDs) (*n* = 19). Data were analyzed by DESeq2. * *p* < 0.05.

**Table 3:** Serum bile acid concentrations.

	Baseline	4 Week Grape	*p* Value
Primary BAs plus conjugates			
CA (nM)	142.9 (228.7)	177.7 (274)	0.98
GCA (nM)	356.5 (691.8)	315.5 (820.8)	0.39
TCA (nM)	60.2 (136.6)	60.2 (228.3)	0.45
CDCA (nM)	200.7 (184.9)	205.4 (194.5)	0.90
GCDCA (nM)	1344.4 (1903.4)	700.5 (920.7)	0.00
TCDCA (nM)	168.8 (231.5)	97.8 (199.5)	0.03
Secondary/Tertiary BAs plus conjugates			
DCA (nM)	485.4 (391.1)	508.6 (422.2)	0.49
GDCA (nM)	491.4 (627.4)	354.8 (466.9)	0.02
TDCA (nM)	50.5 (55.4)	32.4 (54.6)	0.05
LCA (nM)	9.9 (23.6)	6.9 (14.6)	1.00
GLCA (nM)	18.5 (29.6)	14.3 (19.5)	0.41
UDCA (nM)	137 (332.7)	95.9 (169.1)	1.00
Total BAs			
Total	3466.3 (4053.8)	2569.9 (3190.2)	0.04
Ratio: Unconjugated/Total	0.36 (0.22)	0.44 (0.17)	0.21

Values are means (SDs) (*n* = 19).

## Data Availability

Data is contained within the article and supplementary material.

## Supplementary Materials

The following are available online at https://www.mdpi.com/article/10.3390/nu13113965/s1, Table S1: Phytochemicals Analyzed in Freeze-Dried Preparation (value per kg grape powder); Table S2: Nutrient Analysis of Freeze-Dried Table Grape Powder (value per 100 g of grape powder).
