# Supplementary material for: Effect of Standardized Grape Powder Consumption on the Gut Microbiome of Healthy Subjects: A Pilot Study
Source: Nutrients. 2021 Nov 6;13(11):3965. doi: 10.3390/nu13113965 (PMC8619073; doi:10.3390/nu13113965)
Supplement: Supplementary file 1 [file nutrients-13-03965-s001.zip › nutrients-1448942-supplementary.pdf]

Supplementary Table S1: Phytochemicals Analyzed in Freeze-Dried Preparation (value per kg grape powder)

Compounds	Total	Individual
**Catechins**	17 mg/kg	
Catechin		14.6 mg/kg +/- .718
Epicatechin		2.43mg/kg +/- .12
**Anthocyanins**	9.82 mg /kg	
Peonidin		2.62 mg/kg +/- .152
Cyanidin		.24 mg/kg + /- .013
Malvidin		6.96 +/- .298
**Flavonoids**		
Quercetin		1.58 mg/kg +/-.086
**Flavonols**		
Kaempferol		.139 mg/kg +/- .018
Isorhamnetin		.145 mg/kg +/- .016
**Stilbenes**		
Resveratrol		.33 mg/kg +/- .061
**Total Polyphenols** in gallic acid equivalents		354 mg/100g

Supplementary Table S2: Nutrient Analysis of Freeze-Dried Table Grape Powder (value per 100g of grape powder)

**Nutrient**	**Amount (per 100 g powder)**	**Units**
Calories	360	kcals
Total Fat, acid hydrolysis	.87	g
Total Carbohydrate (sugars)	82	g
Protein (N x 6.25)	3.7	g
Calcium	58.7	mg
Iron	1.78	mg
Sodium	32.4	mg
Potassium	1030	mg
Thiamin	.18	mg
Folic Acid	15.5	mcg
Phosphorus	100	mg
Magnesium	36.8	mg
Zinc	.241	mg
Copper	.413	mg
Manganese	.457	mg
Moisture	5.89	g
Ash	3.3	g
